# LTR-Retrotransposons from Bdelloid Rotifers Capture Additional ORFs Shared between Highly Diverse Retroelement Types

**DOI:** 10.3390/v9040078

**Published:** 2017-04-11

**Authors:** Fernando Rodriguez, Aubrey W. Kenefick, Irina R. Arkhipova

**Affiliations:** 1 Josephine Bay Paul Center for Comparative Molecular Biology and Evolution, Marine Biological Laboratory, 7 MBL Street, Woods Hole, MA 02543, USA; frodriguez@mbl.edu (F.R.); awkenefick@ucdavis.edu (A.W.K.); 2 Present address: UC Davis Genome Center-GBSF, University of California, Davis, CA 95616, USA

**Keywords:** retrovirus-like transposable elements, envelope gene (ENV), DEDDy exonuclease, GDSL esterase, dUTPase

## Abstract

Rotifers of the class Bdelloidea, microscopic freshwater invertebrates, possess a highly-diversified repertoire of transposon families, which, however, occupy less than 4% of genomic DNA in the sequenced representative *Adineta vaga*. We performed a comprehensive analysis of *A. vaga* retroelements, and found that bdelloid long terminal repeat (LTR)-retrotransposons, in addition to conserved open reading frame (ORF) 1 and ORF2 corresponding to *gag* and *pol* genes, code for an unusually high variety of ORF3 sequences. Retrovirus-like LTR families in *A. vaga* belong to four major lineages, three of which are rotifer-specific and encode a dUTPase domain. However only one lineage contains a canonical *env*-like fusion glycoprotein acquired from paramyxoviruses (non-segmented negative-strand RNA viruses), although smaller ORFs with transmembrane domains may perform similar roles. A different ORF3 type encodes a GDSL esterase/lipase, which was previously identified as ORF1 in several clades of non-LTR retrotransposons, and implicated in membrane targeting. Yet another ORF3 type appears in unrelated LTR-retrotransposon lineages, and displays strong homology to DEDDy-type exonucleases involved in 3′-end processing of RNA and single-stranded DNA. Unexpectedly, each of the enzymatic ORF3s is also associated with different subsets of *Penelope*-like *Athena* retroelement families. The unusual association of the same ORF types with retroelements from different classes reflects their modular structure with a high degree of flexibility, and points to gene sharing between different groups of retroelements.

## 1. Introduction

Long terminal repeat (LTR) retrotransposons represent a major class of transposable elements (TEs), which move via reverse transcription of the full-length RNA intermediate by the element-encoded reverse transcriptase (RT) [1]. They are structurally similar to vertebrate retroviruses, and undergo the same steps of reverse transcription in their replication cycle [2]. Intracellular LTR retrotransposons typically encode only two genes, the *gag* gene which forms the nucleoprotein core, and the *pol* gene which combines protease, RT, RNase H, and integrase enzymatic activities. Retroviruses additionally code for an *env* (envelope) gene, which endows them with the capacity to interact with cellular membranes for viral entry and exit. The lack of an extracellular stage in the LTR retrotransposon life cycle can be occasionally overcome by capture of an *env* gene from DNA viruses (e.g., baculovirus, phlebovirus, or herpesvirus) [3]. The baculovirus-derived *env* gene in the *gypsy* retrotransposon of *Drosophila melanogaster* has been studied most extensively, revealing infectious and fusogenic properties [4,5,6]. Domestication of *env* genes from endogenous retroviruses has also occurred throughout evolution, giving rise to novel unanticipated host functions [7,8,9].

Bdelloid rotifers are microscopic freshwater invertebrates that reproduce asexually, are highly resistant to desiccation and ionizing radiation, and contain numerous genes of foreign origin in subtelomeric regions [10,11,12]. We previously showed that bdelloid genomes contain canonical LTR-retrotransposons, *Juno* and *Vesta*, forming a deep-branching clade [13], as well as telomere-associated, endonuclease-deficient *Penelope*-like retroelements named *Athena* [14]. Both *Juno* and *Vesta* contain an open reading frame (ORF) 3, which was assumed to code for *env* but revealed no clear-cut homologies to known viral envelope genes.

The genome of the first bdelloid representative, *Adineta vaga*, has been sequenced [15]. Over 8% of its gene content is made up of foreign genes originating from bacteria, fungi, plants, or protists. Known TE families make up to 4% of the 218-Mb assembly, with low copy numbers per family (on average, 1–2 full-length copies and 10 times as many fragments), and high family diversity (over 255 families). Of these, about one-half are represented by retrotransposons, including 24 families of LTR retrotransposons belonging to four clades (*Juno*, *Vesta*, *TelKA*, and *Mag*). Most LTR retrotransposons have transposed recently, as judged by very few or no differences between the two LTRs [15]. Here we focus in detail on their coding capacity, and report that they can code for a variety of extra ORFs of enzymatic origin, which are also found on giant telomeric retroelements called *Terminons* (Arkhipova et al., submitted). We also report that all bdelloid retrotransposon clades, except for *Mag*, carry a dUTPase domain found in certain retroviruses and in basidiomycete LTR-retrotransposons.

## 2. Materials and Methods

### 2.1. Bioinformatics

The annotated *A. vaga* scaffolds containing LTR retrotransposons were downloaded from the genome browser at http://www.genoscope.cns.fr/adineta. Each LTR retrotransposon was manually re-annotated to confirm the presence of intact full-length ORF1, ORF2, and ORF3. Sequences from *P. roseola* (accession numbers DQ985390, EU643489, EU643490) and a natural isolate *Adineta sp*. *11* were also used in the analysis. Homology searches were performed with HHpred (Version HHSuite-2.0.16mod) [16] and visualized with Jalview (Version 2.10.1) [17]. Multiple sequence alignments were done by MUSCLE [18], followed by maximum-likelihood and neighbor-joining phylogenetic analysis, and the resulting trees were edited in MEGA (Version 7.0.18) [19]. Alignments are available from the corresponding author upon request. Coiled-coil motifs were predicted by COILS/PCOILS (Version 2.2) [16], and transmembrane domains with TMHMM (Version 2.0) [20].

For genome-wide analysis, LTR families were extracted from the initial annotation of known *A. vaga* TE families [21]. We estimated the numbers of fragmented copies (longer than 100 base pair (bp), including solo LTRs) and numbers of full-length copies by BLAT (Version 34) [22], using full-length sequences as queries. ORF annotations within each full-length copy were also identified by BLAT search, using family-specific ORF sequences as queries. Alignment of RNA-seq and small RNA reads (NCBI accession Nos. SRP020358 and SRP070765) to the reference genome was performed as in [21]. Aligned sequences were counted for each TE copy and each annotated ORF feature with htseq-count [23].

### 2.2. Nucleic Acid Manipulations

Clonal cultures of *A. vaga* were grown and collected for DNA extraction as described in [15]. We designed the exact-matching forward and reverse primers from the corresponding genomic scaffolds (Table S1) to amplify the full-length ORF3 from each desired element. Polymerase chain reaction (PCR) conditions were as follows: 0.5 U of Q5 High-Fidelity DNA Polymerase (New England Biolabs, Ipswich, MA, USA) in a 25 µL reaction, with 1 µM of each primer, 200 µM dNTPs, 1× Q5 Reaction Buffer and template DNA. Thermocycling parameters were set following the conditions specified in the Q5 High-Fidelity DNA Polymerase manual, with Tm values adjusted for each primer pair. PCR products were electrophoresed in 1.5% agarose gels in 1 × TAE (Tris base, acetic acid, EDTA) buffer, and visualized under UV light. PCR amplicons of the expected size were purified using Wizard^®^ SV Gel and PCR Clean-Up System (Promega, Madison, WI, USA). Prior to T/A cloning, addition of an untemplated dA was done with *Taq* DNA Polymerase (Promega). PCR products were cloned into pGEM-T vector (Promega) and transformed into JM109 (Promega) or DH5a (New England Biolabs) competent cells per the supplier’s specifications. Clones were screened for inserts of the expected size by PCR amplification with the universal primers M13 Forward and M13 Reverse. Plasmid DNA was prepared from selected clones with Zyppy™ Plasmid Miniprep Kit (Zymo Research, Irvine, CA, USA). Templates were sequenced on an Applied Biosystems 3730XL DNA Analyzer at the W. M. Keck Ecological and Evolutionary Genetics Facility at the Marine Biological Laboratory. After inspection of the chromatogram files, the phred/cross_match pipeline [24] was applied to check for quality and to screen out vector sequences. Sequences obtained in this study were deposited in GenBank under accession numbers KY820831–KY820845. Consensus sequences of LTR retrotransposons were deposited in Repbase [25].

## 3. Results

### 3.1. An Overview of LTR Retrotransposon Structure in Bdelloids

**Figure 1:**
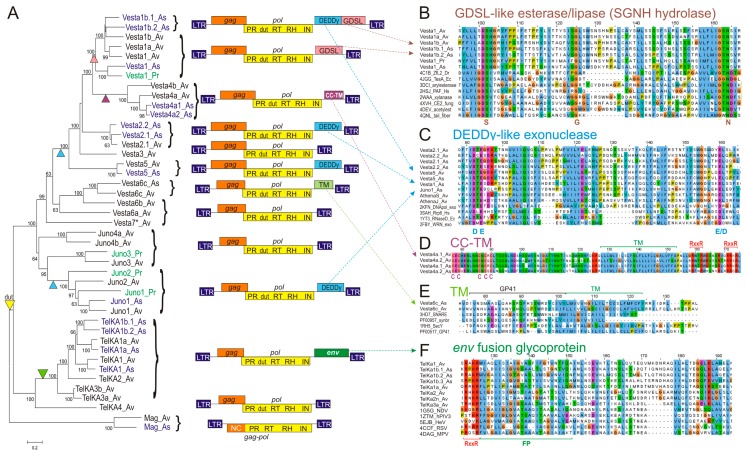
Structure, phylogeny and open reading frame 3 (ORF3) alignments of bdelloid long terminal repeat (LTR) retrotransposons. (**A**) Maximum likelihood phylogram of *pol* genes including protease (PR), dUTPase (dut), reverse transcriptase (RT), RNase H (RH), integrase (IN) domains and the associated ORF structure. Putative ORF3 acquisition/loss events are marked by triangles of matching color. Scale bar, amino acid substitutions per site; (**B**–**F**) Alignments of characteristic regions between retrotransposon ORF3s and selected GDSL esterases/lipases (**B**), DEDDy exonucleases (**C**), transmembrane (TM) proteins (**D**–**E**) and env fusion glycoproteins from paramyxoviruses (**F**). Also shown are catalytic S-G-N residues from SGNH block 3 (**B**), catalytic D/E residues from DEDDy block ExoI (**C**), Cys residues (**D**), TM domains (**D**–**E**), furin-like protease cleavage site (RXXR), and fusion peptide (FP) (**F**).

Of all TE types, LTR-retrotransposons are arguably the easiest to detect and annotate in sequenced genomes due to their characteristic LTR structures. Our recent inventory of the LTR-retrotransposon families in *A. vaga* identified 12 *Vesta*-like families, five *Juno*-like families, six *TelKA*-like families, and one *Mag* family, which in total occupy ~580 kb of genomic DNA [15]. We supplemented this comprehensive dataset with additional LTR retrotransposons from sequenced fosmids from a genomic library of *Philodina roseola (Pr)*, a species from the bdelloid family Philodinidae, which separated from *A. vaga* tens of millions of years ago [26], and from a draft genome of a natural isolate *Adineta sp. 11 (As)*. Notably, not only each congeneric, but also each of the *P. roseola* LTR retrotransposons can be assigned to the corresponding *A. vaga* families (Figure 1A), indicating the early origin of the LTR families and/or extensive horizontal transfer between species.

All LTRs carry TG CA at the ends, vary in length between 159 and 551 bp, and display very few substitutions between two LTRs, which is indicative of recent transposition [15]. As expected, all families code for *pol* genes with a canonical set of enzymatic activities that includes protease (PR), RT, ribonuclease H (RNase H; RH), and integrase (IN), in that order (Figure 1A). Every *gag* gene, except for *Vesta1b*, codes for a typical CX_2_CX_4_HX_4_C Zn-knuckle; in addition, *Mag*, *Juno1*, *Juno2*, and *Vesta6c* code for an adjacent second Zn-knuckle. Curiously, *Vesta1b* not only lacks Zn-knuckles, but also lacks a *gag-pol* translational frameshift, a feature it shares with the *Mag* family. An extra Zn-knuckle upstream of RT in the *pol* gene of all *Juno1* and *Juno2* elements also represents a departure from the standard organization. The GPY/F motif at the integrase C-terminus [27] is present in *Juno1–Juno4* and *Vesta6–Vesta7,* is modified to GPC in *TelKA* and *Vesta6c,* reduced to a proline in *Vesta1–Vesta5*, and is missing from the *Mag* lineage altogether. No chromodomain was found C-terminally to the GPY/F module in any lineage.

Interestingly, all members of the *Juno*, *Vesta*, and *TelKA* families, but not the *Mag* family, contain a dUTPase (dut) domain between PR and RT (Figure 1A), followed by an extra Zn knuckle in *Juno*. The Dut domain often occurs in vertebrate retroviruses, where it can be variably positioned between gag and RT, between RT and IN, or after IN [28,29]. However, it is rarely found in retrotransposons, and has been reported only in basidiomycetes [30], where it is similarly placed between PR and RT.

### 3.2. Types of Acquired Env-Like ORFs

An ORF3 downstream of the *pol* gene is usually assumed to code for an *env*-like protein, as in vertebrate retroviruses. Due to the low conservation of *env* sequences, such assignments often rely on computationally predicted features of broad applicability, such as TM domains, glycosylation sites, protease cleavage sites, or coiled-coil motifs, which are not restricted to *env* genes, but are commonly found in other proteins. Assignment of ORF3 to *env* genes can be unambiguous only when its origin can be traced to another virus [3].

In bdelloids, the *TelKA* clade contains a canonical *env*-like fusion glycoprotein about 600 aa in length, which is most similar to class IF proteins from paramyxoviruses—non-segmented negative-strand RNA viruses such as avian Newcastle disease virus (NDV), human parainfluenza (PIV), respiratory syncytial virus (RSV), metapneumovirus (MPV), and Hendra (HeV) [31] (pfam00523; HHpred alignment over the entire length with E-value = 1.9e^−106^). Regions of high conservation (Figure 1F) include the furin-like protease cleavage site (RXXR), a hydrophobic region (FP, fusion peptide), a trimeric coiled-coil domain, a set of conserved cysteines for disulfide bridge formation between two protease cleavage products, and a C-terminal transmembrane (TM) anchor domain.

In the *Vesta4* clade, a much shorter (220–230 aa) ORF3 lacks detectable homology with known *env* genes, but nevertheless displays two hydrophobic transmembrane regions with a set of cysteine residues in between, followed by an RXXR motif and a coiled-coil domain (Figure 1E). Such structural organization is also suggestive of fusogenic properties, although other functions cannot be ruled out.

A possible *env*-like ORF3 is found in *Vesta6c* LTR retrotransposons from *A. vaga* and a congeneric natural isolate, *Adineta sp. 11* (Figure 1A). This ORF3 is characterized by the presence of TM domains, and an HHpred search reveals weak homology to the retroviral envelope glycoprotein gp41 (PF00517), which mediates fusion with the host cell (*p*-value = 9.2e^−05^) (Figure 1E). In *Adineta*, however, it does not represent a part of the larger gp120-like env precursor, and is instead coded by a small 210–230-aa ORF3. This ORF may be a remnant of an initially present full-length *env*-like ORF.

### 3.3. Unexpected Diversity of Non-Envelope ORF3 Functions

Functional assignment of an ORF3 is often far from straightforward, especially in the absence of a known viral source. For instance, several *copia*-like and *gypsy*-like LTR retrotransposons in plants have long been assumed to code for an envelope-like protein, although it is still unclear if they do [32,33,34,35,36]. Certain plant LTR retrotransposons and vertebrate retroviruses carry extra ORFs with no assignable function [29,37,38]. Surprisingly, we find that bdelloid LTR retrotransposons display a much higher degree of heterogeneity with respect to ORF3 than is typically observed in retroelements.

Use of sensitive HHpred searches allowed us to determine the origin of the remaining extra ORFs, which were previously classified as *env* due to the presence of computationally predicted motifs of broad specificity (TM domains, protease cleavage sites, N-glycosylation sites) [13]. We find that most members of the *Vesta* and *Juno* clades lack bona fide *env* genes, but instead have acquired different ORF3 coding for GDSL esterase/lipase and RNase D-like DEDDy-type exonuclease activities (Figure 1A–C). The DEDDy-type (or DnaQ-like) 3′-5′ exonucleases perform 3′-end processing of various structured RNAs (RNase D, RNase T, exosome subunit Rrp6), but may also act on single-stranded DNAs (WRN, DnaQ, and proofreading subunits of A- and B-type DNA polymerases) [39]. GDSL esterases/lipases are hydrolytic enzymes with broad substrate specificity, named after a GDSL or similar sequence with the catalytic Ser in the first conserved block, and are also designated as SGNH hydrolases, named after the letters specifying the invariant catalytic S, G, N, and H residues in the four conserved blocks [40]. In each of these ORFs, the invariant residues are intact, indicating possible catalytic activity (Figure 1B). Motif DEDD is changed to DEED (Figure 1C). In *Adineta sp*. *11*, both DEDDy and GDSL can occur within a single ORF3 (Figure 1A, top).

In the phylogram on Figure 1A, which depicts currently known families of bdelloid LTR retrotransposons, it may be seen that additional ORF3s, which are family-specific, are notably missing from the earliest branches (*TelKA4*; *Juno3–Juno4*; *Vesta6–Vesta7*). In the more recent branches, the DEDDy-like ORF has been independently acquired at least twice, by *Juno* and by *Vesta* (Figure 1A).

### 3.4. Different ORF3 Types Are Shared between Highly Diverse Retroelements

Interestingly, the diverse ORF3 types (GDSL, DEDDy, CC, TM) are not restricted to LTR retrotransposons. They can also be found in the highly unusual group of bdelloid retroelements which we recently described (Arkhipova et al., submitted). These retroelements, which we call *Terminons*, reveal an extraordinary degree of complexity, coding for multiple diverse ORFs and reaching 40 kb in length. As the principal polymerizing component, they contain *Athena*-like RTs belonging to the enigmatic class of *Penelope*-like retroelements (PLEs) [41]. *Terminons* also harbor a plethora of other ORFs of enzymatic and non-enzymatic nature, which in many families include DEDDy, GDSL, CC-, and TM-containing ORFs.

**Figure 2:**
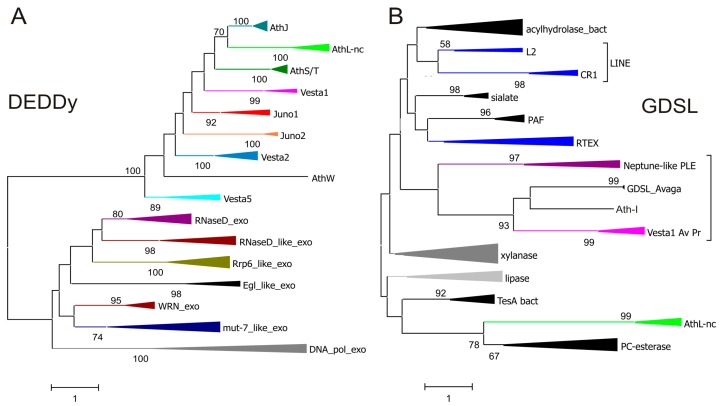
Diverse ORF3 functions in retrotransposons. (**A**) Amino acid sequence similarity between DEDDy-like ORFs from *Juno* and *Vesta* LTR retrotransposons, *Athena* retroelements, and different groups of cellular DEDDy exonucleases from cd09018 sequence cluster in the Conserved Domain Database (CDD); (**B**) GDSL-like ORFs in *Vesta1* LTR-retrotransposons, PLEs, non-LTR (or LINE-like) retrotransposons from esterase-containing clades CR1, L2, and RTEX, and representative groups from the cellular SGNH hydrolase superfamily (cd00229 cluster in CDD). All ORF3 sequences shown in Figure 1 were included and collapsed for better visualization. Branch support values exceeding 50% are shown. Scale bars, amino acid substitutions per site.

We performed phylogenetic analysis of DEDDy-like ORFs from bdelloid retrotransposons, and they are much more similar between the two retrotransposon groups than between TE-associated ORFs and their non-transposable cellular homologs, such as RNase D, mut-7, WRN, Rrp6, and DNA_pol_A exonucleases. Thus, these ORFs are less likely to have been captured from the host than they are likely to have been exchanged between different retroelement types (Figure 2A). This finding hints at the existence of a specialized DEDDy-like ORF pool utilized by diverse retroelements. To some extent, this is also applicable to GDSL-like ORFs: ORFs from *Vesta1* in *A. vaga* and *P. roseola* are apparently related to the GDSL domain in *Athena-I* (Figure 2B), which in turn reveals similarity to a stand-alone GDSL-like ORF in the *A. vaga* host. Due to the absence of catalytic residues in GDSL derivatives from the *Athena-L* family, their origin is more difficult to determine, however, they are consistently grouped with a subfamily of SGNH hydrolases termed PC-esterases (Figure 2B), which are potentially involved in the modification of cell-surface glycoproteins [42]. The esterases found in selected non-LTR retrotransposon clades (L2, CR1, RTEX) [43,44] do not cluster with any of the above ORFs, indicating their independent capture (Figure 2B).

### 3.5. Transcription, Small RNA-Mediated Silencing, and Copy Numbers

**Figure 3:**
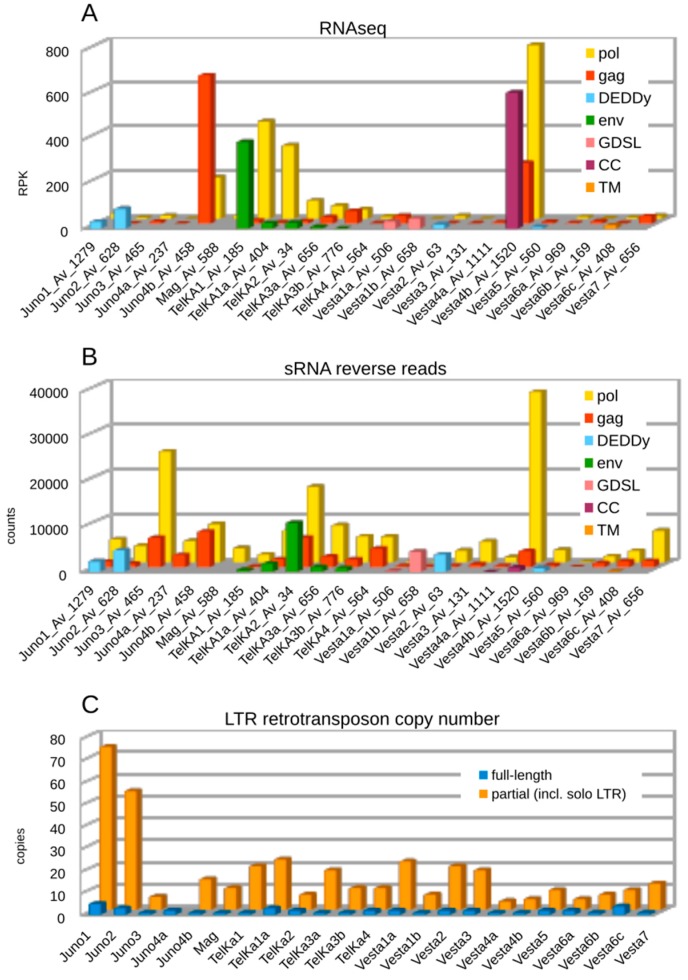
LTR retrotransposon copy numbers, and RNA profiles in *A. vaga*. Distribution of RNAseq reads with RPK (reads per kilobase) values (**A**), and small RNAs in reverse orientation (total counts) mapped to annotated ORFs (**B**) is shown for each family, along with the number of each reference scaffold. Numbers of full-length and fragmented copies (longer than 100 bp) estimated by BLAT, using full-length sequences as queries, are shown in (**C**). ORFs are color-coded as indicated.

In our earlier study investigating transcription and silencing of TE families in *A. vaga*, most LTR retrotransposons were found to be transcriptionally active [21]. However, their expression levels were determined from mapping to full-length TE annotations without subdivision into different ORFs, while ORF3s in LTR retrotransposons typically represent separate transcriptional units, and are expressed from spliced messages. To investigate whether the diverse ORF3s show transcriptional activity, we mapped *A. vaga* transcripts to each ORF individually. The results of RNA-seq profiling are shown in Figure 3A, which displays RPK (number of reads per kilobase) values for each ORF within the *A. vaga* LTR retrotransposon families. In most cases, LTR families display relatively low levels of transcription activity, although there are some notable exceptions. For instance, *Vesta4b* on the scaffold Av_1520 shows high transcript levels within each of the three ORFs (*gag*, *pol*, and CC), possibly reflecting recent arrival of an active element. Interestingly, this scaffold is circularly permuted, which may indicate that it was assembled from an extrachromosomal 1-LTR circle. High transcript levels are also observed for *Juno4b*, which lacks ORF3.

We also investigated whether each ORF type is subject to small RNA-mediated silencing. In *A. vaga*, pi-like small RNAs (sRNA) are preferentially mapped to annotated transposons, with most of the reads being in antisense orientation [21]. Mapping of sRNA read counts by ORF type (Figure 3B) demonstrates that the majority of sRNA reads (66.8%) are mapped to *pol* genes, which occupy most of the TE length. For LTR families with an annotated ORF3 (*env*, DEDDy, GDSL, CC, TM), 22% of sRNA reads are mapped to such ORFs, while 14% are mapped to *gag* and 64% to *pol* gene annotations. For LTR families without an ORF3, *gag* is covered by 29% and *pol* by 71% of the sRNA reads mapped, which is roughly equivalent in terms of read count per kilobase. Comparison of the RNA-seq and sRNA plots shows that transcriptional activity is typically accompanied by sRNA coverage, which involves every ORF type. However, the *env*-containing *TelKA1* and *TelKA1a* show higher levels of transcriptional activity and lower levels of sRNA coverage in comparison with other members of the TelKA clade, which may indicate that their recent arrival has not yet resulted in establishment of a robust piRNA silencing response.

We also attempted to reveal correlations between the presence of ORF3, transcriptional activity, and copy number of LTR retrotransposons. Figure 3C visualizes the number of full-length and partial copies of LTR retrotransposons in each family, with a large proportion of partial copies represented by solo LTRs. While full-length copies are indeed scarce, a DEDDy-like ORF in *Juno1* and *Juno2* might be correlated with a higher overall copy number, however, *Vesta2* and *Vesta5* with the same ORF are low-copy. It is possible that the latter represent earlier arrivals, as evidenced by their more basal position on the phylogenetic tree, and that most of these copies have undergone removal by LTR-LTR recombination, as described in [15].

### 3.6. Sequence Variation in Env-Like and GDSL-Like ORFs

To validate the correct assembly of ORF3 and to evaluate the level of its intraspecific nucleotide sequence variation, we chose the *env*-like ORFs from five *TelKA* families and GDSL-like ORFs from three *Vesta* families for PCR amplification and sequencing. Primers were designed for amplification of full-length ORF3s in each of the families, and the resulting amplicons were cloned and Sanger-sequenced. An additional *env*-like ORF from a non-autonomous family related to *TelKA2*, named *TelKA2n*, which is missing the C-terminal part of *gag* and most of the *pol* gene, was also amplified and sequenced. All families except *TelKA3a* yielded amplicons of the expected length.

The information on sequence polymorphisms is presented in Table 1. In 11 individual 1.7-kb long *env* clones, 16 out of 20 single nucleotide substitutions resulted in amino acid replacement, while in four 1.3-kb GDSL clones, 3 out of 11 substitutions changed the corresponding amino acid. Substitutions which were already present in one of the copies from the genome assembly were marked as “natural”, and a few substitutions were marked as “unique” if they could not be found in the assembly contigs. Most of these mutations apparently reflect natural intragenomic variation, and most of the “unique” substitutions should represent de novo mutations which arose over the five-year period since the genome was sequenced, although a few may still correspond to PCR errors despite the use of the Q5 polymerase with high fidelity exceeding best polymerases by an order of magnitude [45]. If only “natural” variation is considered, we do not find evidence that the number of synonymous substitutions significantly exceeds that of non-synonymous substitutions or vice versa, indicating that intragenomic variation of ORF3s is mostly neutral, and that its level is approximately the same as found in *gag* and *pol* genes (not shown). Indeed, selective forces would be expected to operate during critical steps of the life cycle, such as inter-genomic transmission, while intragenomic *env* evolution is more likely to be neutral. Except for *TelKA1* and *Vesta1*, at least one cloned copy in each family was identical to the full-length reference copy, either at the nucleotide or at the amino acid sequence level. In future experiments, we plan to determine whether the *env*-like or GDSL-like ORFs can exhibit fusogenic or lipolytic properties, respectively.

## 4. Discussion

Our studies uncover an unexpected diversity of additional ORFs in LTR-retrotransposons, which goes beyond their well-known ability to acquire *env* genes from other viruses to facilitate host entry and egress. Earlier studies of plant gypsy-like LTR retrotransposons and animal retroviruses, while revealing extra ORFs, failed to uncover homologies with known proteins, except for two ORFs randomly captured from the host [29,37,38]. In this study, we used sensitive profile-profile searches to detect remote homologs in the HMM profile databases, revealing enzymatic origin for two types of extra ORFs in LTR retrotransposons of microscopic freshwater invertebrates, bdelloid rotifers. It is still unclear whether these ORFs confer proliferative advantages to TEs harboring them, as our analysis of their transcriptional activity did not reveal unambiguous correlations with copy numbers, and their intragenomic evolution does not reveal significant departures from neutrality.

In principle, a DEDDy-like exonuclease might participate in the processing of the 3′-ends of retrotransposon-encoded RNAs, while a GDSL esterase/lipase might facilitate penetration through host membranes during entry and exit. However, the catalytic activity of these ORFs is yet to be demonstrated. A role in post-transcriptional silencing, such as that of *mut-7* in *Caenorhabditis elegans* [46], may also be entertained, although self-limiting TEs would not be expected to survive in the long term, as they would be out-competed [47]. It is also formally possible that enzymatic ORFs may still perform the *env*-like function, despite their diverse origins and the lack of similarities to viral *env* genes. Future experiments aimed at determining fusogenic and/or lipolytic properties of the extra ORFs might help to clarify this issue. However, unlike bona fide *env*-like ORFs in *TelKA*, the GDSL-like and DEDDy-like ORFs lack CC- or TM-domains, suggesting that they do not perform *env*-like functions, but could rather play auxiliary roles in the replication cycle. In selected non-LTR retrotransposons (CR1, RTEX, ZfL2), a catalytically active SGNH hydrolase/esterase, which occupies a *gag*-like position upstream of *pol* and can dimerize via its coiled-coil domain, is thought to play a role in ribonucleoprotein (RNP) assembly and in membrane-dependent transport or localization [43,44]. While DEDDy exonucleases have not yet been reported in retrotransposons, it is worth noting that the metazoan Maelstrom and EXD1 proteins involved in piRNA biogenesis represent catalytically inactive DEDD nuclease derivatives retaining the RNA binding function [48,49]. Maelstrom also contains a Cys-His-Cys motif involved in Zn^2+^ coordination, which can also be noted in *Vesta2* and *Vesta5* DEDDy ORFs.

It is even more perplexing that similar ORFs can be shared between retrotransposable elements of highly diverse nature, such as LTR-retrotransposons and PLEs. Even the esterases from distantly related canonical *Neptune*-like PLEs [50] from fish and mollusks exhibit some similarity, albeit with insufficient clade support (Figure 2B). A plausible explanation for ORF acquisition is the existence of a common step in their transposition cycles permitting RT-mediated template switches in intersecting cellular locations (e.g., sites of RNP assembly). While there is currently no information on the exact transposition mechanisms for complex retroelements, it may be thought that the shared ORF types may be used to confer advantages to different types of retroelements, regardless of specific details of their retrotransposition cycles.

The fact that the extra ORFs are largely detected in the more recent branches of LTR retrotransposons, while missing from the more basal branches, points at a relatively recent acquisition of these ORFs. Another interpretation is that the terminal branches represent recent arrivals and systematically lose extra ORFs, as they become adapted to the intragenomic mode of proliferation. It has been argued that loss of the *env* gene turns endogenous retroviruses into genomic “superspreaders” [51]; however, this is clearly not the case in bdelloids, as is evident from copy number comparisons between *env*-containing and *env*-less families (Figure 3C). LTR retrotransposons in bdelloids are frequently eliminated by LTR-LTR recombination, leading to accumulation of solo LTRs, and by microhomology-mediated deletions, resulting in the formation of partial copies [15]. Thus, acquisition of an *env* gene or its equivalent may be regarded as a path to effective escape, facilitating horizontal mobility. While the role of lipases or exonucleases in this process remains to be determined, it may substitute for the obvious function of envelope genes in unexpected ways, which could be uncovered in future experiments.

## Figures and Tables

**Table 1:** Nucleotide sequence variation in *env*-like and GDSL-like open reading frames (ORFs).

Clone	Reference Scaffold/Contig ^1^	Substitutions, bp	Substitutions, aa	Natural aa Differences	Unique aa Differences
*env1*	1591/5150	4	4	R-Q, E-Q, T-I	I-V
*env1a.1*		3	2		I-T, V-A
*env1a.4*	1200/4393	0	0		
*env1a.8*		3	3	V-A, T-I	S-F
*env2.1*	34/303	0	0		
*env2.2*		2	1	I-T	
*env2.3*		4	3	V-I, I-T	M-I
*env2n.1*	680/3155	1	1		A-S
*env2n.2*		2	1		D-G
*env3b.1*	776/3459	0	0		
*env3b.2*		1	1	I-T	
*ves1*	494/2540	8	3	T-S, R-S, H-Q	
*ves1a*	506/2575	1	0	silent	
*ves1b.1*	658/3084	1	0	silent	
*ves1b.3*		1	0	silent	

^1^ Scaffold numbering: http://www.genoscope.cns.fr/adineta (annotated), and Contig numbering: WGS shotgun assembly CAWI000000000.2 (unannotated); One reference scaffold/contig is listed for each family.

## Supplementary Materials

The supplementary materials are available online at www.mdpi.com/1999-4915/9/4/78/s1. Table S1. Primers used for ORF3 amplification.
