# Supplementary material for: LTR-Retrotransposons from Bdelloid Rotifers Capture Additional ORFs Shared between Highly Diverse Retroelement Types
Source: Viruses. 2017 Apr 11;9(4):78. doi: 10.3390/v9040078 (PMC5408684; doi:10.3390/v9040078)
Supplement: Supplementary file 1 [file viruses-09-00078-s001.pdf]

Table S1. Primers used for ORF3 amplification.

Primer name[1]	Sequence (5′→3′)
Vesta1a_F	TGGCCATCTTGTCCATCTGT
Vesta1a_R	AAGCGAAACAGAAAGGAAAAGT
Vesta1b_F	ACATCTTCCCACCTTTCCTCA
Vesta1b_R	CAGAAGTGATAGCATCGAGTGT
Vesta1_F	GCATTTCTGTGTTTGTTACTTCCT
Vesta1_R	AGGAAACGCGTAACCCAAAT
TelKA1_F	AACGATTCGGAACAAAATGC
TelKA1_R	TGGACCGTTGTTGTTGATTC
TelKA1a_F	GCATTAGACATGCAGAATCATATC
TelKA1a_R	CAAGTTAAAGATCGGAAAGCAA
TelKA2_F	GACGACGTCAACCAGCAGTA
TelKA2_R	TGCTTGACGTCTTTTTGGAA
TelKA2n_F	CAAGCAAAAGAAGCAGTCCA
TelKA2n_R	TGGTTGTTGAGTGTGTTCGAT
TelKA3a_F	TCACAAACATGTTACAACTCTTCA
TelKA3a_R	ACGCTCTCCTGAGCGTCTTA
TelKA3b_F	TCACAAATATGTCGAAATTCTTCG
TelKA3b_R	GGGTAGGTGGGGAGAGAGAG

[1] F: forward; R: reverse
